# Adaptive capacity to reduce disaster risks in informal settlements

**DOI:** 10.4102/jamba.v16i1.1488

**Published:** 2024-06-07

**Authors:** Khulekani E. Ndabezitha, Betty C. Mubangizi, Sokfa F. John

**Affiliations:** 1 NRF/SARChI Chair in Sustainable Rural Livelihoods, School of Management, IT and Governance, College of Law and Management Studies, University of KwaZulu-Natal, Durban, South Africa; 2 Centre for Mediation in Africa, Political Sciences Department, Faculty of Humanities, University of Pretoria, Pretoria, South Africa

**Keywords:** adaptive capacity, disaster risk, eMalahleni Local Municipality, informal settlements, resilience

## Abstract

The eMalahleni Local Municipality (eLM) in Mpumalanga province, South Africa, has a number of informal settlements because of the influx of people seeking employment in the municipal area. These informal settlements are exposed to a number of hazards, including underground fires, air and water pollution, sinkholes, abandoned mining areas and acid mining drainage. South Africa’s National Development Plan (NDP) incorporates the United Nation’s Sustainable Development Goals, which are intended to upgrade informal settlements on suitable land. The Department of Human Settlement recognised the gap in the policy because upgrading only included physical structures and did not include adaptive capacity for communities to create resilience to withstand disasters. The researcher used a case study research design for the inquiry intended to recommend adaptive capacity and reduce disaster risks in informal settlements in the eLM. Purposive sampling was used to select 25 participants from eLM, provincial government departments and informal settlements. The data were analysed using thematic analysis based on the study’s conceptual framework. The research findings revealed that the government has not done much to involve vulnerable communities during the development of policies to reduce disaster risks within informal settlements. In particular, the failure of the government to promote and reinforce public participation in disaster risk reduction programmes leaves the vulnerable communities defenceless.

**Contribution:**

This study strengthens the intergovernmental structures and public participation to reduce disaster risks in communities. This study discourages a silos mentality and encourages coordination between government departments to identify root causes by applying the pressure and release model for effective disaster risk reduction.

## Introduction

Throughout the world, informal settlements pose a significant challenge for governments, highlighting their struggle to address the escalating influx of individuals migrating to urban areas in pursuit of improved living standards (Marutlulle 2017). As the United Nations (UN) (2018) observed, a staggering one-quarter of the global urban population, comprising 883 million people, resides in informal settlements, with 520 million concentrated in Asia. Within sub-Saharan Africa, over half of urban residents inhabit such settlements characterised by hazardous living conditions (Zerbo, Delgado & González 2020). These settlements are marked by overcrowding, looming threat of eviction, inadequate infrastructure including poorly constructed roads and insecure housing lacking access to essential services such as water and sanitation, thus exacerbating residents’ vulnerability to life-threatening diseases (Matamanda, Dunn & Nel 2022; Zerbo et al. 2020). Amid these challenges, the concept of ‘adaptive capacity’ emerges as a crucial consideration, reflecting the community’s ability to respond and adapt to changing circumstances, policies and environmental conditions, ultimately shaping their resilience in the face of adversity.

**FIGURE 1:**
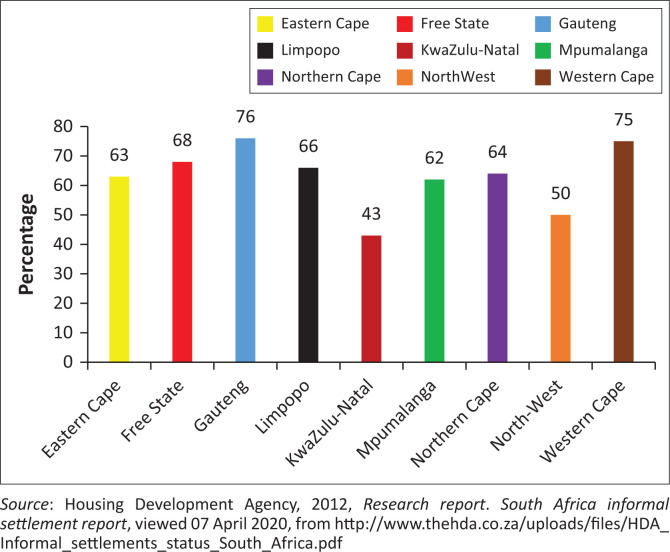
Number of informal settlements not in a backyard per province.

In line with the principles outlined in the Constitution of the Republic of South Africa (1996), which enshrines the provision of basic services as a fundamental right for all citizens, South Africa grapples with the persistent challenge of meeting the needs of its populace (hereafter called the Constitution). Section 26 of the Bill of Rights expressly guarantees ‘everyone’s right to access adequate housing and essential services such as healthcare, food, water, and social security’ (Constitution 1996). However, despite these constitutional guarantees, informal settlements persist, reflecting a gap between policy aspirations and on-the-ground realities. The 2011 census data, as reported by the Housing Development Agency (2012), indicates that 64% of informal structures are standalone buildings, with an additional 7% constructed in the backyard of formal dwellings. These data underscore the prevalence of informal settlements, whether as standalone structures or as annexes to formal housing. Amid this landscape, the concept of adaptive capacity emerges as crucial, highlighting the resilience of informal settlement communities in navigating and responding to the challenges they face, including limited access to basic services and inadequate housing conditions. Recognising and enhancing the adaptive capacity of these communities are essential for promoting their ability to thrive and effectively cope with evolving socio-economic and environmental conditions. The 2012 statistics of the Housing Development Agency is the most appropriate to this study and is the latest official report. Figure 1 presents a breakdown of standalone informal settlements in South Africa per province.

eMalahleni Local Municipality (eLM) has experienced a high influx of people seeking a better life. This influx caused a huge backlog of 55 390 of government provided houses (eLM 2019). The eLM and Department of Human Settlements (DoHS) have adopted a policy in 2019 for the upgrading of human settlements in response to the huge backlog and number of disaster risks posed by the 30 714 households that live in 71 informal settlements in eLM (2019). These disaster risks include underground fires, sinkholes and air and water pollution. This policy deals only with the construction of the formal structure of a house and is not a policy of adaptive capacity focused on building resilience to withstand any disaster caused by hazards in informal settlements. This study applied the disaster pressure and release (PAR) model to assess the informal communities’ participation level in policy development and implementation for disaster risk reduction. Furthermore, to evaluate the role of government and non-government institutions in reducing vulnerability through hard and soft engineering measures to create adaptive capacity to reduce disaster risks in informal settlements.

The term ‘adaptation’ is found in the National Climate Change Adaptation Strategy of the Department of Environmental Affairs (2019:9), which defines adaptive capacity as the ‘ability of the system, institutions, humans and other organisms to adjust to potential damage to take advantage of opportunities or to respond to consequences’. Hence, this study aimed to generate information for the informal communities’ and municipal systems’ ability to adapt to potential disaster risks and return to normal in the event of disaster.

### Previous attempts to address informal settlements in South Africa

Huchzermeyer (2008) posited that eradicating or eliminating informal settlements in South Africa is unattainable, given the projected high rate of new informal settlements estimated by the UN. Instead, they advocated for a proactive approach by the South African government to upgrade existing informal settlements. Huchzermeyer suggested that local authorities develop strategies and plans to transition informal settlements from a vulnerable status quo to a resilient and improved social environment.

Sibiya, Aigbavboa and Thwala (2013) identified various barriers to upgrading informal settlements in Gauteng Province, South Africa, including delays in decision-making by the DoHS management, legal challenges such as litigation and court orders, limited participation of informal settlement communities because of the top-down approach of the DoHS and inconsistencies in government processes. On 21 August 2008, the South African Constitutional Court ruled that the Gauteng government must upgrade the informal settlement Joe Slovo instead of evicting its residents (Huchzermeyer 2008). This Constitutional Court judgement was deemed a victory for informal settlement communities nationwide.

Given the persistent growth of informal settlements and the significance of the Constitutional Court ruling, this research aims to explore ways to develop adaptive capacity for disaster risk reduction as a means to upgrade the eLM informal settlements from hazardous environments to safer ones.

Satterthwaite (2017) found that most of the measures to reduce disaster risks within a particular jurisdiction are the sole responsibility of the local government and rely on its competence to address issues of concern within its jurisdiction.

### Disaster pressure and release model

In 2004, the PAR model gained international recognition as a pivotal framework for understanding vulnerability dynamics and advancing disaster risk reduction efforts (Wisner et al. 2014). Renowned in academia and community-based organisations, the PAR model empowers communities to identify their vulnerabilities and strengths, rather than relying solely on external interventions for disaster risk management solutions (Wisner et al. 2014). This study utilises the PAR model to delve into the vulnerability factors impacting residents of informal settlements, proposing adaptive capacity measures to bolster resilience against disaster risks. Van Niekerk (2005) and Anderskov (2004) affirmed the PAR model’s efficacy in analysing disaster scenarios, while Gohl’s (2008) study in Mozambique and Awal’s (2015) research in Bangladesh demonstrated its utility in assessing vulnerability and guiding risk reduction strategies. Notably, Awal’s study shed light on the PAR model’s aptness in understanding vulnerability progression amidst climate-related disasters. Furthermore, Mabaso (2019) highlighted the PAR model’s significance for disaster management practitioners, underlining its role in quantifying vulnerability and devising effective interventions. Central to the PAR model are the concepts of Progression of Safety for Vulnerable Communities, which addresses root causes and reduces pressure, and Community-based Disaster Risk Reduction (CBDRR), focusing on hazard mitigation and achieving safe conditions. The following paragraph will explore these key aspects to underscore the model’s value in enhancing adaptive capacity within informal settlements.

### Progression of safety for vulnerable communities

**FIGURE 2:**
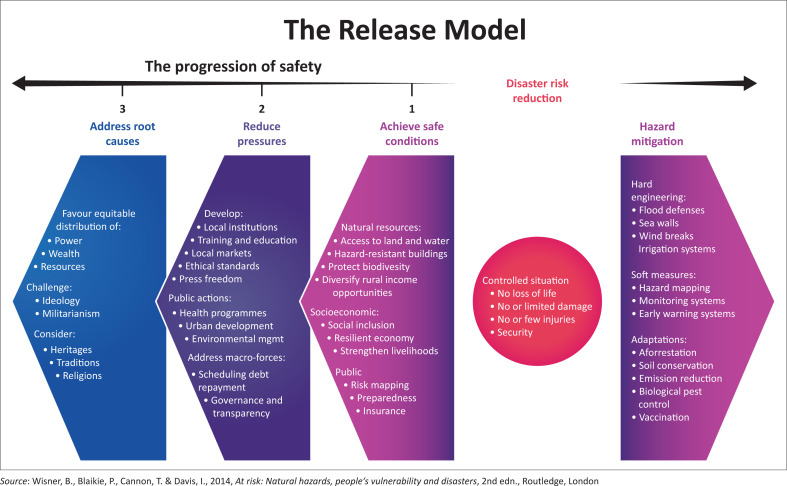
Disaster pressure and release model: Progression of safety.

The progression of safety for vulnerable communities involves deliberate actions to identify and mitigate hazards to transition from vulnerability to safety. This process encompasses various activities, including hazard mitigation and management, achieving safe conditions, reducing pressure, addressing root causes for disaster risk reduction, building adaptive capacity and creating adaptation strategies for disaster risks (Figure 2). These activities necessitate collaboration among stakeholders from diverse backgrounds. As highlighted by Twigg (2015), effective disaster risk reduction requires multidisciplinary partnerships that facilitate sharing resources, ideas and budgets for optimal outcomes. This collaborative approach signifies a shift from viewing disaster risk reduction as solely a government problem to acknowledging it as a shared responsibility within public administration, as emphasised by Clark-Ginsberg (2020) and Twigg (2015). This collaborative effort involves community members, ward councillors, disaster management practitioners, research institutions (universities and the private sector), government departments and local authorities (Phiri, Van Nikerk & Van Eeden 2016). Engaging communities in disaster risk reduction activities is crucial as it enables them to take ownership of their environment’s safety measures, as Nahayo et al. (2017) noted.

In addition, community involvement enhances awareness of hazards and strengthens coping capacities (Nahayo et al. 2017). Stakeholders need to listen to and integrate indigenous knowledge into disaster risk reduction strategies during their interactions with communities, as highlighted by Iloka (2016). A successful progression of safety requires incorporating indigenous knowledge and scientific information to ensure comprehensive and effective risk-reduction efforts (Iloka 2016). Through this collaborative and inclusive approach, stakeholders can work together to achieve meaningful progress in enhancing the safety and resilience of vulnerable communities.

### Community-based disaster risk reduction approach

According to Wisner et al. (2014), the progression of safety within the PAR model emphasises public safety through risk mapping, preparedness and insurance as essential components of effective disaster risk reduction. Gaillard and Maceda (eds. 2009) highlight the pivotal role of community involvement as a prerequisite for achieving sustainable disaster risk reduction and ensuring ownership of government interventions in the progression of safety. This community involvement is encapsulated in the CBDRR approach (eds. Gaillard & Maceda 2009). Risk mapping, a crucial aspect of CBDRR, entails the analysis of hazards, vulnerabilities and coping capacities using scientific methodology (Daddoust et al. 2018). Given the complexity of risk mapping involving scientific methods and advanced technology, it is incumbent upon local authorities and other stakeholders to simplify the process to facilitate full community participation. Liu et al. (2018) underscore the significance of community participation in risk mapping, considering it as the most effective approach for gathering accurate disaster risk information and devising tailored risk reduction strategies for vulnerable communities. The CBDRR approach offers benefits by enhancing understanding of hazards and fostering preparedness for response during disaster events.

In our study framework, these insights align closely with our focus on understanding the progression of safety within informal settlements. By emphasising public safety through risk mapping and community involvement, our research seeks to explore the role of stakeholders, including local authorities and community members, in simplifying risk mapping processes and promoting full community participation. Moreover, our study aims to leverage the CBDRR approach to enhance understanding of hazards within informal settlements and foster a sense of preparedness among residents. Through this framework, we aim to contribute to the discourse on effective disaster risk reduction strategies tailored to the unique needs of vulnerable communities within informal settlements.

## Research methods and design

For this study, the researcher chose a qualitative research approach to conduct the study with the intention of answering the research questions and fulfilling the study objectives. The qualitative research approach is associated with studying human behaviour, interaction, emotions, livelihoods and cultural beliefs (Creswell 2014; De Vos et al. 2011; Thompson 2018; Tracy 2019). Therefore, the researcher chose a qualitative research approach to study communities living in vulnerable conditions in informal settlements and their adaptation to capacity to reduce disaster risk. The researcher conducted face-to-face semi-structured one-on-one interviews with the participants and observed all the following COVID-19 protocols: Wearing a face mask, using sanitiser and keeping a social distance of 1.5 m. According to De Vos et al. (2011), research designs within the qualitative research approach can be narrative biography, ethnography, phenomenology, grounded theory and case study. For this study, the researcher selected the case study research design for the enquiry intended to create adaptive capacity and reduce disaster risks in informal settlements in the eLM. The case study design was selected because it allowed the researcher to conduct an in-depth study on the complex issues concerning a group of people in a specific area, time and real-life context (Algozzine & Hancock 2017; Crowe et al. 2011).

### Description of the study area

The study was conducted in the eLM. This local municipality is located in the Nkangala District Municipality, Mpumalanga province 25.8891° S, 29.2320° E. The study area derives its name from the Nguni word eMalahleni, which translates to ‘place of coal’. The eLM is on the boundary of the City of Tshwane, the City of Ekurhuleni and the City of Johannesburg of the Gauteng province.

eMalahleni Local Municipality has experienced a high influx of people seeking employment in the power generation stations and business establishments. This influx of people has increased the number of informal settlements in the study area. The eLM currently consists of 71 informal settlements and some of these informal settlements are located in hazardous environments with no plans for relocation despite a 10–15 year project approved in the municipality’s Integrated Development Plan (IDP) (eLM 2019). Some of these informal settlements are located in areas with the following hazards: below the flood line, underground fires, water and air pollution, built above the servitude of gas pipes and built on abandoned mining areas (eLM 2019). The study was conducted in the following informal settlements: Old Coronation, Thabo Mbeki, Kamgewana, Spring Valley, Sizanani, Emgodini and Emsagweni (KwaGuqa Ext.1).

### Sampling and targeted population

This study employed a non-probability sampling approach to select participants, specifically utilising a purposive sampling method. The choice of this sampling method was deliberate and aligned with the research objectives (Daniel 2012). Purposive sampling allowed the researcher to select participants based on specific criteria relevant to the study’s focus. In this case, the participants were purposefully selected from the eLM in the Mpumalanga province of South Africa. The selected sample comprised seven community members; one eMalahleni Informal Settlement Cluster worker, seven community development workers; three ward councillors; one eLM: Disaster Management Centre worker; one eLM: Safety, Security and Law Enforcement worker; one eLM: Spatial Planning, Land Use Management and Urban Renewal Unit worker; one eLM: Human Settlement Unit worker; one eLM: Environmental and Waste Management worker; one Provincial DoHS worker and one Provincial Department of Cooperative Governance and Traditional Affairs (DCoGTA): Disaster Management Centre worker. These participants were chosen as they were directly involved in issues related to informal settlements, ensuring that they could provide relevant data aligned with the study’s objectives. The purposive selection of participants enabled the research to capture a comprehensive range of perspectives and insights pertinent to the study’s focus on informal settlement issues within the eLM.

### Data collection tools

The qualitative research approach offers flexibility in utilising multiple data collection tools within a single study (Aurini, Heath & Howells 2016; Olsen 2012). These tools encompass various methods such as in-depth interviews, participatory observation, focus groups, questionnaires and accessing secondary data sources, including hard-copy and online documents such as journals, books, organisational strategic plans, frameworks, theses, conference papers and even corpus analysis (Aurini et al. 2016; De Vos et al. 2011; Olsen 2012; Rose, McKinley & Baffoe-Djan 2019). In this study, the researcher opted to employ in-depth interviews and secondary data as primary sources of information. These choices were made deliberately based on their suitability for capturing rich, contextual insights into the phenomenon under investigation. The use of in-depth interviews facilitated a nuanced exploration of participants’ perspectives and experiences, while secondary data sources provided additional context and background information. By employing these specific data collection methods, the researcher aimed to gather comprehensive data that would contribute to a deeper understanding of the research topic.

### Description of the participants

The participants were categorised into government officials, community members and ward councillors. The following subsections briefly describe these two groups: government community participants and ward councillors.

#### Government officials

The researcher appreciated the honesty of the government officials in pointing out the limitations and weak points of the current administration system. Table 1 gives a brief description of the government officials who took part in this study.

**TABLE 1:** Description of the government officials.

Name	Organisation	Directorate of the eLM
Participant 1	eMalahleni Informal Settlement Cluster	Communications
Participant 2	eLM	Disaster Management Centre
Participant 3	eLM	Safety, Security and Law Enforcement
Participant 4	eLM	Spatial Planning, Land use Management and Urban
Participant 5	eLM	Human Settlement Unit
Participant 6	eLM	Environmental and Waste Management
Participant 7	Provincial DoHS	Planning Unit
Participant 8	Provincial DCoGTA	Disaster Management Centre

eLM, eMalahleni Local Municipality; DoHS, Department of Human Settlements; DCoGTA, Department of Cooperative Governance and Traditional Affairs.

#### Community member and ward councillors participants

During the data analysis, the researcher observed the similarities in the community members’ responses about the provision of basic services and their experience of communities in the informal settlements of the eLM. There were similarities observed in the ward councillors responses about the provision of basic services which projected a positive picture regarding the service delivery contrary to community members assertion. Table 2 gives a brief description of the community and ward councillor’s participants.

**TABLE 2:** Description of the community and ward councillor’s participants.

Name	Settlement	Designation
Participant 9	Old Coronation	Community member
Participant 10	Old Coronation	Community development worker
Participant 11	Old Coronation	Ward councillor
Participant 12	Thabo Mbeki	Community member
Participant 13	Thabo Mbeki	Community development worker
Participant 14	Kamgewana	Community member
Participant 15	Kamgewana	Community development worker
Participant 16	Spring Valley	Community member
Participant 17	Spring Valley	Community development worker
Participant 18	Spring Valley	Ward councillor
Participant 19	Sizanani	Community member
Participant 20	Sizanani	Community development worker
Participant 21	Emgodini	Community member
Participant 22	Emgodini	Community development worker
Participant 23	Emsagweni (KwaGuqa Ext.1)	Community member
Participant 24	Emsagweni (KwaGuqa Ext.1)	Community development worker
Participant 25	Emsagweni (KwaGuqa Ext.1)	Ward councillor

### Ethical considerations

Ethical clearance to conduct this study was obtained from the (University of KwaZulu-Natal Humanities and Social Sciences Research Ethics Committee) (No. HSSREC/00001939/2020).

## Results

### Findings concerning public participation and consultation for policy development of disaster risk reduction

The researcher attempted to discover how the informal settlement communities participate in policy development and implementation for disaster risk reduction in the informal settlements in the eLM. The participants shared their experiences regarding public participation and consultation in policy development and implementation to prioritise disaster risk reduction in the informal settlements.

The participants had different feelings and experiences concerning public participation and consultation, particularly for disaster risk reduction. The sentiment of all community members was that there is no public participation and consultation concerning policy development and implementation for disaster risk reduction, especially for vulnerable communities living within informal settlements. One community member stated that ‘eLM conduct community outreach for Integrated Development Plan [*IDP*] but not for policy development and implementation to reduce disaster risks in the informal settlements’ (Participant 12). Most community members were frustrated that community outreach programmes for IDP meetings only took place within formalised settlements and that the communities from the informal settlements are asked to join in those formalised settlements’ meetings. The community members were further frustrated because during community outreach IDP meetings, the voices of the informal settlement communities are silenced when they are told that the municipality will begin an informal settlement upgrade when there is land available.

All government participants conceded that the government has not done much to involve vulnerable communities during the development of policies to reduce disaster risks within informal settlements. An eLM response official stated that ‘consulting the communities during the policy development is time-consuming, but it has great benefits to inform policy direction and incorporate indigenous knowledge’ (Participant 1). All participants confirmed that there are no public participation and consultation to develop policies meant to reduce disaster risks and create adaptive capacity within the informal settlements of the eLM.

#### Hard engineering measures

All the participants agreed that there is no consultation about hard engineering measures to be introduced to mitigate and eliminate the risk of disasters occurring in the informal settlements. Most community members indicated that there are no consultations to introduce hard engineering measures such as flood defence lines, firebreaks, lightning conductors, flycatchers or any other hard engineering measures by government or NGOs for communities living in the informal settlements. One community member stated that:

‘[*I*]t is difficult to receive any intervention from the government since there is no proper community engagement with informal settlement communities to raise possible hard engineering measures to address the needs of the informal settlements.’ (Participant 14)

Most community members were frustrated because there is no consultation about the proposed hard engineering measures as a result of poor consultation between the government and the informal communities. Most community members indicated that they need simple hard engineering measures that will not cost millions of rand to make the informal settlements safer to live in, but that it is difficult to get these measures in place as there is no consultation between government and communities. For example, the community members mentioned that one minor hard engineering measure needed is something to kill mice as they feed on dumpsites and make their way into informal houses to destroy belongings. An eLM response official stated, ‘the main objective of the government is to make land available and move the communities living in the informal settlement to served land with proper infrastructure’ (Participant 4).

The community participants made many suggestions based on their knowledge for possible hard engineering measures that can be introduced to create adaptive capacity within the informal settlements. Unfortunately, these suggestions cannot be explored because no one in the government is willing to listen to and engage with the communities in the informal settlements.

#### Soft engineering measures

All the participants indicated that there is no consultation and public participation to raise possible soft engineering measures for informal settlements. Two of the eight government participants conceded that there is some level of hazard mapping and monitoring by government institutions but that there is minimal introduction of soft engineering measures to mitigate the disaster risks. One community member stated that:

‘[*G*]overnment authorities are fully aware of the hazards in the informal settlement but they are not will[ing] to engage us and work with us to eliminate or adapt to such hazards while they are working on the long-term solution.’ (Participant 21)

All the community members agreed that hazard mapping and monitoring by government institutions must be followed by intervention measures such as early-warning systems to alert the communities of danger.

The community members are frustrated with the poor public participation and consultation for possible soft engineering measures to adapt to the hazardous environment while the government is working towards a long-term solution. The community members and government participants had different views about the possible soft engineering measures. Most community members (16 of 17) stated that soft engineering measures are not solely based on technology but also on the use of indigenous knowledge. However, all the government participants believed that the lack of infrastructure within the informal settlements disadvantaged communities living there from receiving soft engineering measures to develop adaptive capacity to reduce disaster risks. An eLM response official stated that:

‘[*T*]the government is moving towards the Fourth Industrial Revolution (4IR) which is the digital revolution, and most of the soft engineering measures are enabled by technology, which is not feasible within informal settlement due to the lack of technological infrastructure.’ (Participant 1)

Both government participants, community members and ward councillors, had similar ideas and possible solutions to reduce disaster risks in the informal settlements. However, these ideas can only be merged when there is healthy public participation and consultation to formulate workable solutions for informal settlement communities. The common sentiment indicated that while the people living in the informal settlement are characterised by hazards, it is not too insurmountable to identify workable soft engineering measures that will create adaptable capacity to reduce disaster risks while the government is exploring long-term solutions.

## Discussion of findings concerning public participation and consultation for policy development of disaster risk reduction

The literature revealed that it is paramount for communities to be involved in the formation of hazard mitigation plans during public participation outreach programmes to ensure cooperative governance (Hemingway & Gunaway 2018; ed. Paleo 2009). It is evident from the literature that public participation and consultation are critical for the formulation of policies to reduce disaster risks in the community environment (Hemingway & Gunaway 2018; ed. Paleo 2009). Chapter 4 of the *Municipal Systems Act* (MSA) (2000) also states that the local sphere of government should have public participation and consultation on matters concerning governance. Section 16(1)(b)(i) of the MSA states that the municipality must develop a culture of municipal governance to build the capacity of the local community. The participants suggested that there is no public participation and consultation concerning the policy development and implementation for disaster risk reduction, especially for vulnerable communities in informal settlements. This suggests that the administration of the eLM is acting contrary to the provision of Section 16 of the MSA. One participant stated that the eLM conducts community outreach programmes for the IDP but not for policy development intended to reduce disaster risks in vulnerable communities.

The National Disaster Management Framework (NDMF) calls for the intentional promotion and reinforcement of disaster risk reduction programmes through the various communication platforms to ensure public participation and that disaster risk reduction programmes are communicated to all stakeholders, especially the communities directly affected by disaster risks (South Africa 2005). The feedback from the participants suggested that there are no public participation programmes meant for disaster risk reduction, especially in the communities directly affected by disaster risks in the informal settlements.

### Hard engineering measures

The literature of Vidrikova et al. (2017) indicated that the government is responsible for ensuring that there are hard engineering measures to eliminate disruption to community infrastructure and economy. The literature further suggested that communities must play a major role in public participation and consultation concerning the hard engineering measures for the mitigation of vulnerability and they must be involved (Hemingway & Gunaway 2018; ed. Paleo 2009). Furthermore, the literature suggests that involvement of communities during in the hard engineering measures prioritisation and implementation because it helps communities take ownership of any hard engineering measures for disaster risk reduction.

Rehak, Slivkova and Brabcova (2017) suggested that functional hard engineering measures in communities are a basic right to safety and prosperity of communities at large. Furthermore, Pescaroli and Alexander (2016) suggested that any community without hard engineering measures is physically and economically vulnerable. Various studies by Abubakari and Twum (2019); Gupta and Barman (2017); Pescaroli and Alexander (2016) revealed that the majority of human deaths are caused by the absence of hard engineering measures that expose communities to disaster risks. The feedback from the participants suggested that there is no consultation regarding the implementation of hard engineering measures to eliminate and mitigate disaster risks in the informal settlements of the eLM. Furthermore, their feedback suggested that the absence of hard engineering measures in the informal settlements exposes the communities to physical and economical vulnerability to any type of disaster risk. One of the participants indicated that it is impossible to receive any hard engineering measure from the government as there is no proper consultation with communities to raise possible hard engineering measures to address the specific needs of the informal settlements. The community participants indicated that the communities have suggestions for possible hard engineering measures projects that will not cost millions of rand but these suggestions cannot be communicated to the administration because there is no health consultation platform for disaster risk reduction purposes. Some of the hard engineering measures suggested by the participants include flood defence lines, lightning conductors and fly and mice traps. The literature revealed that the absence of hard engineering measures in communities affects their livelihood and adaptive capacity to transit from progression of vulnerability to safety (Pescaroli & Alexander 2016).

The failure of the government to promote and reinforce public participation in disaster risk reduction programmes leaves the vulnerable communities defenceless. The feedback from the participants suggested that the administration of the eLM fails to facilitate healthy consultation on possible hard engineering measures, eliminating the opportunity for the community to raise possible hard engineering measures suitable to the informal settlements’ needs and informed by indigenous knowledge systems.

### Soft engineering measure

The literature revealed that hard engineering measures alone are not sufficient for effective disaster risk reduction and that soft engineering measures are critical to complement hard engineering (Mechler 2016; ed. Paleo 2009). The literature further suggested that education, training, public awareness, hazard mapping, early-warning systems and land use planning are critical to create resilience and adaptive capacity in vulnerable communities (Mechler 2016; ed. Paleo 2009). Furthermore, Nahayo et al. (2017) revealed that communities should be involved through consultation to create ownership and partnership with the government in the effort to create adaptive capacity in the vulnerable communities. Public participation and consultation in disaster risk reduction matters involving soft engineering measures must ensure cooperative governance between the government and communities. The feedback from the participants suggested that there is no healthy cooperative governance between the government and communities in relation to consultation about soft engineering measures for vulnerable communities. The feedback from the participants indicated a gap that the eLM administration should improve by consulting communities concerning soft engineering measures.

The United Nations International Strategy for Disaster Reduction (UNISDR) has been championing for the prioritisation of soft engineering measures through public participation and consultation for effective disaster risk reduction interventions, and Priority 3 of the Sendai Framework for Disaster Risk Reduction (SFDRR) (2015–2030) (South Africa 2002) encourages governments and NGOs to invest in disaster risk reduction for resilience programmes based on both soft and hard engineering measures. *The Disaster Management Act*, no. 57 of 2002 (South Africa 2002) and NDMF encourage the local sphere of government to increase local capacity in order to minimise disaster risks through public participation in vulnerable communities. The participants suggested that the administration of the eLM is contravening the Disaster Management Act and the NDMF. Public participation is critical for vulnerable communities living in informal settlements to build trust and adaptive capacity to reduce disaster risks. The participants revealed that there is an indication of hazard mapping by the government, but there has been no consultation with the communities to propose soft engineering measures to mitigate the hazards identified during the mapping. This means the government is failing to inform the communities and allow them to be part of the solution to mitigate risks. The government participants indicated that the absence of hard engineering measures is because of the nature of informal settlements, which are unplanned and informal. Therefore, there is no hard infrastructure to enable the implementation of soft engineering measures for disaster risk reduction and the creation of adaptive capacity for the progression of safety. The government participants’ comments showed a lack of willingness to build adaptive capacity to reduce disaster risks in the informal settlements.

The feedback from participants suggested that with proper consultation, possible soft engineering measures can be proposed despite the fact that there is no hard infrastructure in the informal settlements. The feedback from the participants suggested that possible soft engineering measures can be proposed through the indigenous knowledge systems because the most effective measure is cooperative governance between government and communities. The participants suggested that parallel ideas can only be merged through healthy consultation and public participation on matters concerning creating adaptive capacity in the informal settlements of the eLM. The feedback from the participants outlined the serious gap that the eLM administration should consider rectifying to improve consultation with communities.

## Contribution of the study

The PAR model provides two types of progression, namely the progression of safety and vulnerability. The progression of safety includes the following practical and systematic factors: to address root causes, to reduce pressures, to achieve safer conditions and to mitigate hazards. These four factors are useful to inform and guide administrations in effective disaster risk reduction. The contribution of the study is to strengthen the intergovernmental structures in all spheres of government to ensure effective integration and coordination of activities to reduce disaster risks in communities. This study discourages a silos mentality and encourages coordination between government departments to identify root causes by applying the PAR model to effective disaster risk reduction.

The study can be useful during the review of the National Development Plan (NDP) 2030 and DoHS upgrade policy to incorporate the progression of safety and adaptive capacity to reduce disaster risks in informal settlements. The study’s findings suggest that the DoHS upgrade policy should not only focus on structural upgrades but also consider the adaptive capacity of the informal communities to be resilient when disaster strikes. In the context of local government administration, the study makes a contribution by taking into account the development of safety and making provisions for supporting the implementation of the construction of adaptive capacity for informal settlements within the jurisdiction.

## Conclusion

Hemingway and Gunaway (2018); Paleo (ed. 2009) suggested that it is important for communities to be involved during the development of hazard mitigation plans through public participation and consultation programmes to ensure cooperative governance. The UN frameworks and South African legislations advocate for public participation of and consultation with communities on matters related to disaster risk reduction activities. Chapter 4 of the MSA (2000) requires the local sphere of government to facilitate public participation and consultation on matters concerning governance. Furthermore, Section 16(1)(b)(i) of the MSA (2000) specifies that municipalities must develop a culture of municipal governance to build the capacity of local communities. In addition, the NDMF (South Africa 2005) requires the spheres of government to intentionally promote and reinforce public participation through various communication platforms to ensure that communities receive disaster risk reduction programmes and projects well. However, the feedback from the participants suggested that there is no public participation and consultation on policy development and implementation for disaster risk reduction for vulnerable communities living in informal settlements.

## References

[CIT0001] Abubakari, M. & Twum, K.O., 2019, ‘Cities and floods: A pragmatic insight into the determinants of households’ coping strategies to floods in informal Accra, Ghana’, *Jàmbá: Journal of Disaster Risk Studies* 11(1), 1–14. 10.4102/jamba.v11i1.608 PMC6407457 30863511

[CIT0002] Algozzine, B. & Hancock, D., 2017, *Doing case study research: A practical guide for beginning researchers*, Teachers College Press, New York, NY.

[CIT0003] Anderskov, C., 2004, ‘Anthropology and disaster: An analysis of current trends within anthropological disaster research, and an attempt to construct an approach that facilitates theory building and applied practices: Analysed with vantage point in a case study from the flood-prone Mutarara District in Mozambique’, Master’s thesis, University of Aarhus.

[CIT0004] Aurini, J.D., Heath, M. & Howells, S., 2016, *The how to of qualitative research: Strategies for executing high quality projects*, Sage, London.

[CIT0005] Awal, M.A., 2015, ‘Vulnerability to disaster: Pressure and release model for climate change hazards in Bangladesh’, *International Journal of Environmental Monitoring and Protection* 2(2), 15–21.

[CIT0006] Clark-Ginsberg, A., 2020, ‘Disaster risk reduction is not “everyone’s business”: Evidence from three countries’, *International Journal of Disaster Risk Reduction* 43, 101375. 10.1016/j.ijdrr.2019.101375

[CIT0007] Constitution of the Republic of South Africa, Act 108 of 1996, 1996.

[CIT0008] Creswell, J.W., 2014, *A concise introduction to mixed methods research*, Sage, California.

[CIT0009] Crowe, S., Cresswell, K., Robertson, A., Huby, G., Avery, A. & Sheikh, A., 2011, ‘The case study approach’, *BMC Medical Research Methodology* 11(1), 1–9. 10.1186/1471-2288-11-100 21707982 PMC3141799

[CIT0010] Daddoust, L., Khankeh, H., Ebadi, A., Sahaf, R., Nakhaei, M. & Asgary, A., 2018, ‘The social vulnerability of older people to natural disasters: An integrative review’, *Health in Emergencies and Disasters Quarterly* 4(1), 5–14. 10.32598/hdq.4.1.5

[CIT0011] Daniel, J., 2012, *Sampling essentials: Practical guidelines for making sampling choices*, Sage, California.

[CIT0012] Department of Environmental Affairs, 2019, *National climate change adaptation strategy*, viewed 25 April 2020, from https://cer.org.za/wp-content/uploads/2019/05/DEA-Draft-climate-change-adaptation-strategy.pdf.

[CIT0013] De Vos, A., Strydom, H., Fourche, C. & Delport, C., 2011, *Research at grassroots*, 4th edn., Van Schaik, Pretoria.

[CIT0014] eMalahleni Local Municipality, 2019, *Integrated development plan 2019–2020*, viewed 08 April 2020, from https://www.emalahleni.gov.za/v2/documents/category/122-idp.

[CIT0015] Gaillard, J.C. & Maceda, E.A. (eds.), 2009, ‘Participatory three-dimensional mapping for disaster risk reduction’, in *Participatory learning and action*, pp. 109–118.

[CIT0016] Gohl, S., 2008, ‘Local governance and disaster risk management in Mozambique’, Master’s dissertation, University of the Western Cape.

[CIT0017] Gupta, M.R. & Barman, A., 2017, ‘Socio-economic perspective of disaster risk reduction and recovery–understanding socio-technological means of sustainability’, in *Managing higher education for sustainability and livelihood*, vol. 1.

[CIT0018] Hemingway, R. & Gunawan, O., 2018, ‘The natural hazards partnership: A public-sector collaboration across the UK for natural hazard disaster risk reduction’, *International Journal of Disaster Risk Reduction* 27, 499–511. 10.1016/j.ijdrr.2017.11.014

[CIT0019] Housing Development Agency, 2012, *Research report. South Africa informal settlement report*, viewed 07 April 2020, from http://www.thehda.co.za/uploads/files/HDA_Informal_settlements_status_South_Africa.pdf.

[CIT0020] Huchzermeyer, M., 2008, ‘South Africa’s approach to eradicating informal settlements: An urgent call for change’, *Trialog* 98, 48–53.

[CIT0021] Iloka, N., 2016, ‘Indigenous knowledge for disaster risk reduction: An African perspective’, *Jàmbá: Journal of Disaster Risk Studies* 8(1), 272. 10.4102/jamba.v8i1.272 29955294 PMC6014035

[CIT0022] Liu, W., Dugar, S., McCallum, I., Thapa, G., See, L., Khadka, P. et al., 2018, ‘Integrated participatory and collaborative risk mapping for enhancing disaster resilience’, *ISPRS International Journal of Geo-Information* 7(2), 68. 10.3390/ijgi7020068

[CIT0023] Mabaso, N., 2019, ‘Evaluation of disaster risk reduction initiatives at eThekwini. Municipality’s disaster management unit’, Master’s dissertation, UKZN.

[CIT0024] Marutlulle, N., 2017, ‘Causes of informal settlements in Ekurhuleni Metropolitan Municipality: An exploration’, *Africa’s Public Service Delivery and Performance Review* 5(1), 1–11. 10.4102/apsdpr.v5i1.131

[CIT0025] Matamanda, A.R., Dunn, M. & Nel, V., 2022, ‘Broken bridges over troubled waters: COVID-19 and the urban poor residing in Dinaweng informal settlement, Bloemfontein, South Africa’, *South African Geographical Journal* 104(3), 309–327. 10.1080/03736245.2022.2028669

[CIT0026] Mechler, R., 2016, ‘Reviewing estimates of the economic efficiency of disaster risk management: Opportunities and limitations of using risk-based cost–benefit analysis’, *Natural Hazards* 81(3), 2121–2147. 10.1007/s11069-016-2170-y

[CIT0027] Municipal Systems Act, no. 32 of 2000, 2000.

[CIT0028] Nahayo, L., Mupenzi, C., Kayiranga, A., Karamage, F., Ndayisaba, F., Nyesheja, E.M. et al., 2017, ‘Early alert and community involvement: Approach for disaster risk reduction in Rwanda’, *Natural Hazards* 86(2), 505–517. 10.1007/s11069-016-2702-5

[CIT0029] Olsen, W., 2012, *Data collection: Key debates and methods in social research*, Sage, California.

[CIT0030] Paleo, U.F. (ed.), 2009, *Building safer communities: Risk governance, spatial planning and responses to natural hazards*, vol. 58, Ios Press, Amsterdam.

[CIT0031] Pescaroli, G. & Alexander, D., 2016, ‘Critical infrastructure, panarchies and the vulnerability paths of cascading disasters’, *Natural Hazards* 82(1), 175–192. 10.1007/s11069-016-2186-3

[CIT0032] Phiri, A., Van Nikerk, D. & Van Eeden, E.S., 2016, ‘Theoretical orientation of community based disaster risk management’, *Global Journal of Human-Social Science* 16(1), 1–15.

[CIT0033] Rehak, D., Slivkova, S. & Brabcova, V., 2017, ‘Evaluation the resilience of critical infrastructure subsystems’, in M. Cepin & R. Bris (eds.), *Safety and reliability–theory and application*, pp. 955–962, CRC Press, Florida.

[CIT0034] Rose, H., McKinley, J. & Baffoe-Djan, J.B., 2019, *Data collection research methods in applied linguistics*, Bloomsbury Academic, New York, NY.

[CIT0035] Satterthwaite, D., 2017, ‘The impact of urban development on risk in sub-Saharan Africa’s cities with a focus on small and intermediate urban centres’, *International Journal of Disaster Risk Reduction* 26, 16–23. 10.1016/j.ijdrr.2017.09.025

[CIT0036] Sibiya, M. Aigbavboa, C. & Thwala, W.D., 2013, ‘Barriers to informal settlements upgrading in the Gauteng province of South Africa’, 2nd international Conference on Innovations in Electrical and Civil Engineering, Pattaya, December 17–18, 2013.

[CIT0037] South Africa, 2005, *National disaster management framework*, Government Printer, Cape Town.

[CIT0038] South Africa, 2002, *Disaster Management Act, no. 57 of 2002*, Government Printers, Pretoria.

[CIT0039] Thompson, R., 2018, ‘A qualitative phenomenological study of emotional and cultural intelligence of international students in the United States of America’, *Journal of International Students* 8(2), 1220–1255. 10.32674/jis.v8i2.144

[CIT0040] Tracy, S.J., 2019, *Qualitative research methods: Collecting evidence, crafting analysis, communicating impact*, John Wiley & Sons, New Jersey.

[CIT0041] Twigg, J., 2015, *Disaster risk reduction*, Overseas Development Institute, London.

[CIT0042] United Nations, 2018, *World urbanisation prospects*, viewed 06 July 2020, from https://population.un.org/wup/Publications/Files/WUP2018-Report.pdf.

[CIT0043] Van Niekerk, D., 2005, ‘A comprehensive framework for multi-sphere disaster risk reduction in South Africa’, Doctoral dissertation, Thesis–PhD, NWU, Potchefstroom.

[CIT0044] Vidrikova, D., Boc, K., Dvořák, Z. & Řehák, D., 2017, *Critical infrastructure and integrated protection*, Association of Fire and Safety Engineering, Ostrava.

[CIT0045] Wisner, B., Blaikie, P., Cannon, T. & Davis, I., 2014, *At risk: Natural hazards, people’s vulnerability and disasters*, 2nd edn., Routledge, London.

[CIT0046] Zerbo, A., Delgado, R.C. & González, P.A., 2020, ‘Vulnerability and everyday health risks of urban informal settlements in Sub-Saharan Africa’, *Global Health Journal* 4(2), 46–50. 10.1016/j.glohj.2020.04.003

